# Defining what’s at stake: a person-centered approach to conceptualizing the health and social impacts of police violence in the United States

**DOI:** 10.3389/fpubh.2025.1591186

**Published:** 2025-10-14

**Authors:** Jé Judson, Mienah Z. Sharif

**Affiliations:** ^1^Division of Epidemiology & Community Health, School of Public Health, University of Minnesota, Minneapolis, MN, United States; ^2^Department of Social, Behavioral, and Population Sciences, Tulane University School of Public Health and Tropical Medicine, New Orleans, LA, United States; ^3^Division of Community Health Sciences, School of Public Health, University of California, Berkeley, Berkeley, CA, United States

**Keywords:** police violence, structural racism, structural violence, critical race theory, life course perspective, abolition, intergenerational effects, health equity

## Abstract

The increasing efforts among public health researchers to examine the connections between police violence and health outcomes has resulted in growing discoveries about the implications for both direct and vicarious exposure as well as disparities by race and ethnicity. To date, the conceptualization of police violence and health has largely focused on single causes and/or mechanisms at one point in time and focused on individuals most proximal to impact. However, the prevailing conceptualizations are limited in scope. They are relatively linear, do not account for multiple dimensions of harm, and are void of temporal factors that span across communities and generations–all factors that are sustained by forms of structural racism. We offer a reconceptualization guided by the Public Health Critical Race Praxis (PHCRP), a public health offshoot of Critical Race Theory, that offers public health professionals a framework and semi-structured process for centering racism in their analyses and implications of police violence on health. Our conceptualization is supported by multiple case studies, and we conclude with concrete recommendations for public health professionals to draw on as strategies to address police violence and advance health equity.

## Introduction

Within the last decade, public health researchers have increasingly examined connections between exposure to one or more forms of police violence and myriad negative health outcomes in the United States (US). This includes an increased risk for depression, stress, or trauma (1–3), metabolic disorders (4, 5), and negative birth outcomes (6, 7). These implications have been found for both direct victims of policing and those exposed vicariously to the violence, and they have disparate impacts by race and ethnicity of respondent.

As part of the vast undertaking to examine and address police violence, particularly its racialized nature, multiple conceptual models and frameworks have been developed. Most focus on the “how” of racialized policing, that is, the causes and mechanisms of exposure with emphasis on the complex web of attendant health impacts. Some conceptualizations also incorporate the underlying “why,” that is, the etiology of racialized policing, including the structures and policies involved. Each has their own strengths and shortcomings.

Alang and colleagues largely set the tone, taking an accessible and race-conscious approach to describe five primary pathways for how police brutality impacts Black health (1). These pathways account for (1) fatal injuries (i.e., increased mortality), (2) increased morbidity through adverse chronic health, (3) the stress of racist public reaction, (4) criminal-legal, medical, and funeral costs to freedom and financial stability, and (5) disempowerment that results from the collaboration of oppressive structures. It is one of the few conceptualizations to explicitly address the health strain that results from the public racism of community and media responses to brutality or to detail specific causes of economic strain. A subsequent analysis by Alang and colleagues added an intersectional lens to center the mechanisms of gendered racism related to police violence (8). Overall, this framework operates more as an overview, upon which we aim to elaborate in more detail.

DeVylder and colleagues take a more mechanism-based approach using a structural-psychological lens (9). Their work is grounded in the understanding that anti-Black racism is an antecedent to the current structure of policing, and they develop a model of police violence that applies to other populations, specifically other minoritized groups. Their model is composed of the structural risk and protective factors that influence police exposure, factors that heighten the potential for escalation into violence, and broadly, the physical and mental health effects, other health related costs, and impediments to health recovery. Each section details more subcategories of conditions or factors researchers can and should consider. This is an excellent model for understanding at a high level, the pathway for how police violence functions. It, however, does not include a temporal aspect of how harm permeates nor does it delve into the details and nuances of how health impacts manifest and function in relation to those larger structures.

Simckes and colleagues similarly take a mechanism-based approach that lends itself to statistical modeling, however, their contextual analysis is more criminologically rooted. Heavy emphasis is placed on the necessity and difficulty of policing, citing broader safety concerns, while their historical contextualization consists primarily of describing neighborhood racial and socioeconomic composition (10). Absent is the connecting structural analysis for how and why these factors are related. In this way, this model falls into the common trap of interchanging race with racism, losing how racism is fundamentally embedded within the structuring, policies, and (racially targeted) practices of policing as well as the creation of the same “disadvantaged” neighborhoods that legitimize the need for public safety officers. This model is oriented to the notion that *improper* policing creates adverse health, lending itself to a more reformist vantage point, where calls to action might include more investments into the institution of policing. Moreover, while the pictorial form of their model depicts short- and long-term health effects across socioecological level and temporal elements, their conceptualization is underdeveloped, lacking a discussion of how these effects manifest simultaneously and interact across levels, and their cyclical pathways only summarily mentioning how people are interconnected and unfortunately reemphasize their central focus on criminality (i.e., hypersurveillance leads to more crime, which leads to more policing).

Each of these framework-models adds a critical component to the conversation about how police violence impacts health, yet essential elements addressing the wide dispersion of harm within and across the networks people belong to, including temporal factors, are still inadequately accounted for. To address this, we present a person-centered approach to reconceptualizing the health impacts of police violence, illustrated by four case studies of Black women and girls killed by police. We offer a process for thinking through the multidimensional scope of destruction to illuminate the true magnitude of harms (re)produced by police violence over time. We integrate important prior conceptualizations and frameworks, building out the threads introduced by Alang et al. (1), and introduce a model for considering police violence like an earthquake rather than a static (and isolated) event. Moreover, we aim to address how existing frameworks minimize the compounding effects of grief, time, and re-traumatization and demonstrate how public health professionals can account for the degree to which multiple people are impacted over time and the same people are repeatedly impacted. Our goal is to provide a guide for how public health designs future studies, frames conclusions, and more importantly, informs actions to reverse these trends such that recommendations do not reinforce the same structures and processes that created this cascade of violence.

## Theoretical groundwork

### Public health critical race praxis

We find it is necessary to ground our reconceptualization with a shared theoretical starting point, and offer a framework guided by the Public Health Critical Race Praxis (PHCRP). The PHCRP is a public health offshoot of Critical Race Theory and offers public health professionals a framework and semi-structured process that “combines theory, experiential knowledge, science and action” to inform race-conscious work and advance racial equity. PHCRP provides healthcrits four focus areas to work through in any research endeavor including: (1) Contemporary Patterns of Racial Relations—the acknowledgment of the pervasive and insidious nature of racism within the study’s socio-political context (e.g., the “ordinariness of racism” in society), (2) Knowledge Production—the recognition of the role racism plays within the research process itself (i.e., theories, methods) and the attempt to disrupt this by challenging dominant norms and practices in the research process, (3) Conceptualization and Measurement—the integration of Focus areas 1 and 2 allowing researchers to advance their study design (e.g., inclusion of race conscious constructs and/or variables), and (4) Action—the real world implementation of findings such that they do not merely benefit the researchers and/or the research paradigm. Research should be conducted with the goal of drawing on findings to shift inequitable power dynamics and ultimately improve conditions for the communities directly impacted by the research question(s). Each focus area has specific corresponding CRT-derived principles (e.g., intersectionality, disciplinary self-critique, ordinariness of racism) (11).

### Focus area 1: primacy of racism—policing is a racialized project

Any conceptualization of police violence must be grounded in an historical perspective. Policing in the US is unequivocally undergirded by white supremacist and white nationalist goals. This has been extensively discussed in different bodies of literature, but in brief, policing in the US originates in the late 1700s in the form of slave patrols. Much of the research on policing begins in the 1830s with the creation of the Boston police force, which was the first policing entity to be publicly funded. By the 1890s formalized policing existed across the US (12). Irrespective of funding and formal titles, it is critical to note that across time, the core function of policing has persisted: to excessively monitor, control, and dehumanize Black and other minoritized populations. As migration patterns evolved over time, different configurations of policing emerged (i.e., Immigration and Customs Enforcement, or ICE) to reinforce racist immigration “policies” that determine who is, and is not, worthy of asylum or citizenship rights. Racism is the cornerstone of US policing.

### Focus area 2: knowledge production

When viewing policing as both rooted in and a component of structural racism, it becomes clearer how conceptualizations inadvertently understate the violence it produces. Existing public health frameworks tend to address police violence as a discrete phenomenon producing specific health outcomes and social implications across levels of the social ecology with varying degrees of interaction. This approach lends itself to the generation of statistical models, with certain factors treated as potential mediators, moderators, or confounders to a health outcome of interest. Yet, this approach distances researchers from those enduring the violence. People are observed as anonymous data points, becoming a collection of ailments and misfortunes (i.e., “Black bodies”) divorced from the totality of their experiences.

Conventional framing also arbitrarily confines outcomes to be produced from “time of event” (i.e., a specific act of violence) forward, where the event is primarily defined as extraordinary actions of “wrongdoing” (e.g., excessive force) rather than emerging from the more permanent state of structured police aggression. It operates from an unspoken assumption that prior to the designated event, that people were “normal” and that they are now changed due to the specific event chosen by the researcher despite all the unknown and unnamed acts of police violence prior to and in between the period of study. This event-based approach (13) limits what can be known about the complexity and interconnectivity between the lives of people impacted by police violence. Many impacts are simply not considered in traditional statistical models, while others that are more nebulous and difficult to measure either receive cursory treatment or are omitted entirely in interpretations of findings, limitations, or future directions. Erasure and oversimplification of the health implications of police violence is the result.

Alternatively, a person-centered approach begins to counteract these traditional approaches of depersonalized understandings of policing emanating from either nameless datapoints in aggregate or martyrized individuals whose stories largely stop being their own as they are co-opted by the media. By centering people and the routine lives they led, the complexity of harm sustained becomes more evident. It illuminates how other people, communities, systems, and structures interact simultaneously to both passively and actively exacerbate police violence, how this accumulation of harm persists and operates through time, and how it is compounded by the myriad ways Black and minoritized people already experience dehumanization via racial capitalism. Moreover, when looking beyond the martyrized names to additionally spotlight the multitude of unspoken names, it becomes more difficult for researchers to lose themselves in explaining or legitimizing the systems that produce these harms and clarifies the objective of correcting and repairing.

### Focus area 3: conceptualizing and measurement

When you begin to center people’s experiences, both in the first person or through secondary accounts, in research endeavors, it becomes evident that it would be more accurate to conceptualize police violence as a continuous process of mass violence rather than a series of discrete incidents. Police violence shifts the very fabric of how a population thinks about themselves, interacts with others, and engages with the world. The shift happens in a manner that is both calculable and intangible, while the distribution of harm is far wider and more prolonged than is currently articulated in extant epidemiological literature. Some have argued that the nature of US policing constitutes genocide against Black and other minoritized communities (14). Policing involves not only killing, but in collaboration with other social structures and the state, it destabilizes specific groups of people, forcibly separating and destroying families to ensure sustained physical and psychological harm (15, 16). This continuous threat of violence operates across the life-course; it is both intergenerational and transgenerational.

One could argue that existing public health frameworks with broad domains of causes and consequences of police violence (e.g., premature mortality) provide enough flexibility to extrapolate additional conditions. However, within these domains there are nuances and insidious experiences that would be difficult to conceive of without having directly experienced this stigmatized and racially directed form of violence, particularly when research efforts primarily rely on aggregate-level data. What is not explicitly named, cannot be measured nor incorporated into more traditional models for conceptualizing the causes and consequences of US policing, let alone endeavoring to intervene.

### Focus area 4: action

In this paper, we concretize these larger, more abstract concepts by centering the stories and narratives of named individuals who have experienced these forms of violence. We can never fully know what each person nor the lives interconnected to their own experience without exhaustively speaking to each person over time, but we can start to infer the collection of stressors they experience and the systems they would have to encounter, by connecting what can be gleaned through secondary sources to what is already understood about the various structures and systems that individuals encounter in the realm of social services, healthcare, education, and so forth. By starting with the people directly affected, it more clearly highlights all of what is contained in each domain and how these pathways intersect for the individuals at present, their networks, and the future generations who will learn about policing largely before they ever have the potential to experience it.

## Methods

### Approach

Our discussion centers on four Black women and girls (infant) killed between 2009 and 2019: Marquita Bosley, Alteria Woods, Seyaira Stephens, and Atatiana Jefferson. We draw on data from two prior studies to construct these case studies. The first underlying study examined the causes and consequences of 573 cases of fatal policing against Black women & girls over 20 years. This study addressed (1) why the police encounter originally occurred, (2) how the encounter unfolded, (3) and health and circumstantial factors that were salient to the encounter (17). In the second underlying study, one case was selected (Alteria Woods) for a discourse analysis of all media coverage pertaining to her case to understand how it influenced public perception and legal ramifications. Additionally, interviews were conducted by the first author with two of Alteria’s relatives to understand how the media atmosphere impacted the family (18). Taken together, the facts of each case as presented in the various underlying documentation (articles, reports, etc.) and the statements of family members, alongside existing conceptual frameworks, are synthesized to create our reconceptualized model for thinking about the totality of individual-level, systems-level, and generational impacts of police violence.

These four individuals were chosen intentionally to illustrate a range of underlying causes for deadly policing, victim’s life circumstances, and experiences of the network of people connected to these decedents. There is nothing more egregious about their stories compared to others in the larger sample yet given the relative underexposure of Black women victims of police brutality in the media, we chose more recent cases, cases that had more news coverage of their deaths to draw upon more details, and Alteria specifically because we were able to speak directly with her family.

### Data

The cases from the initial underlying study were located through the *Fatal Encounters* dataset with supporting information located through internet (Google) and database (NewsBank and NexisUni) searches of the decedent’s name and names of relevant witnesses and survivors. This documentation included news articles, police reports, city reports, legal documents, and blog posts. For Alteria Woods’ case, the first author contacted the family through an email address included on a memorial website. After several conversations over email, both the younger sister and mother expressed a desire to have their perspective included, and after obtaining approval by Tulane University Social-Behavioral IRB [2021–1,158], both were interviewed. Each interview was conducted by the first author over Zoom and lasted approximately 75 min. Ahead of each interview, participants were emailed a copy of the consent form, and before beginning we reviewed the consent document together after which they verbally consented to that study, the reuse of information in subsequent studies, as well as to the use of their real names throughout published materials. Given the public facing nature of the situation, their anonymity would not have been possible. Moreover, they each expressed a desire to have their unaltered recollection of events in the public record.

### Analysis

For the original analysis of the 573 Black women and girls, we used qualitative description (QD) to address who was involved, under what circumstances, and to what ends for each case including the decedent and those connected to the event (17). We divided the original case information in the *Fatal Encounters* dataset among a team of three coders and appended the supporting documentation (*n* = 332) for coding and analysis. We iteratively read through articles following leads and links to other sources until we were able to address all underlying research questions (17). Direct quotes from families and witnesses come from this collection of news articles and related documentation.

For the interviews we used narrative inquiry. A semi-structured guide was created to prompt discussion into the family’s recollection of how they were notified about Alteria’s death, how they interacted with law enforcement and media, and how their community did or did not show up for them. However, participants had the space to narrate the aspects of the story they felt were important, with follow-up questions asked as they became relevant to confirming details and clarifying the timeline of events. Direct quotes from both Yolanda and Alexus Woods come from these interviews.

## Reconceptualizing the production of harm: four case studies

**Figure 1:**
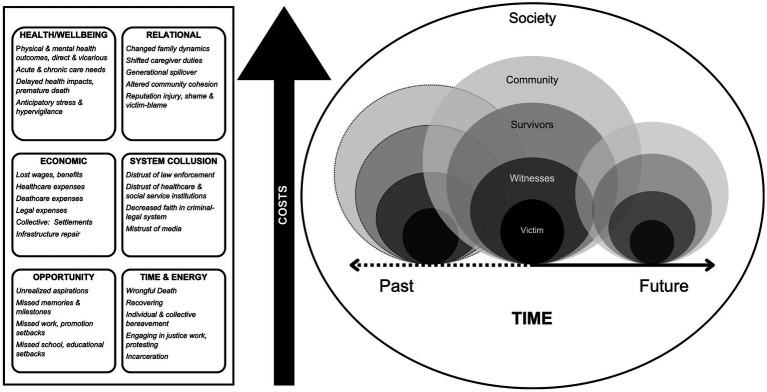
Person-centered conceptual framework of the ballooning costs of police violence layered across communities and time.

We begin our reconceptualization for elaborating the harms of police violence with Figure 1. Much like an earthquake, police violence emanates from a point in time, or an epicenter of direct violence. For each direct victim of police violence, there are also impacts on witnesses, surviving loved ones, the community (local and broader), and society at large that, like the aftershocks of an earthquake, the consequences and timing of which are unknown. This is related to the concept of “linked lives” (19), and we apply this to police violence to describe how people are interconnected across contexts and time such that we are each impacted by others’ experiences and conditions in the present, past, and concerns for the future. These categories are not intended to be rigid and mutually exclusive, as surviving loved ones and unfamiliar community members may also be witnesses to the violence. They are guides for how to think through the web of people impacted by one death. While our discussion focuses on police fatalities, we emphasize that other non-fatal acts of police physical, sexual, psychological, and neglectful violence are no less impactful in peoples’ lives and on community health.

People are also nested within and influenced by the conditions of their society, which we will not detail. In short, people are impacted both indirectly and directly by factors such as racialized capitalism that maintains the privileges of some at the cost of sustaining the oppression of and harms done to others. For the sake of visual clarity, the figure only includes three victim-centered points, but at any given time there are multiple acts of police violence perpetrated against one or more individuals that impact many people and communities concurrently. There are additionally lingering impacts from past events and the anticipation of future impacts.

The magnitude of harm (associated costs), indicated by the size of the circles, will vary case by case. The costs we have categorized and listed to the left of the figure are by no means exhaustive, but in addition to the well-described health impacts addressed in prior conceptual frameworks, we expand onto the relational, economic, and systems impacts that are underdeveloped, and add “Opportunity” and “Time & Energy” costs that have, to date, been overlooked or excluded. Focusing on the four cases, we discuss the mounting costs across domains, within and across nested relationships, and over time, to exemplify the breadth of destruction produced by police violence. Among the nearly 1,400 people killed during police encounters each year (20) these names are likely unfamiliar to most. Yet, it is this relative anonymity of victims that obscures the depth and breadth of the impact of police violence. We uplift these four women and girls and propose this framework to directly confront this pattern.

### Victims

Marquita Bosley was a woman from Oakland, California and 25 years of age. In August 2009, she was driving with her almost two-year-old son in the car to his father’s house. Nearby, police were attempting to stop another driver on the road for “erratic driving.” When the driver of that car did not pull over, the police pursued him, leading to a high-speed chase through a residential neighborhood. The fleeing driver crashed into Marquita, killing her, and injuring her son (21). Marquita was a collateral victim of a police encounter that emerged over an unnecessary chase that endangered the public. These such chases disproportionately impact Black communities and Black women (17, 22).

Seyaira Stephens was a one-year-old infant from Baton Rouge, Louisiana, riding in the car with her mother, grandmother, and several other adults and children in October 2017. An off-duty officer was speeding through the area at 94 mph and crashed into their vehicle, ejecting Seyaira from the vehicle. She succumbed to her injuries after 2 weeks in the hospital (23). Seyaira was a victim of police negligence, an under-examined form of police violence that contributed to 7% of the fatalities of Black women and girls across a 20-year period (17). We conceptualize negligence not only as a failure to act or adhere to protocol while on duty, but abuse of power, which in this case involves institutional collusion to shield officers from consequences even when in clear violation of the law they are entrusted to uphold.

Alteria Woods was a 21-year-old woman from Gifford, FL, asleep at the home of her boyfriend (Andrew Coffee IV) in March 2017, when officers conducted a pre-dawn no-knock drug raid at the house in search of her boyfriend’s father (Andrew Coffee III). Coffee IV heard what he thought was someone breaking into his bedroom window and reached for his gun, firing shots as police launched flash-bang grenades into his room (24). In response to Coffee IV’s gunfire, police unloaded their weapons, shooting Alteria 10 times and killing her (25). Alteria is the collateral victim of an ongoing and ineffective war on drugs that militaristically pursues low-level drug offenders in their homes and other densely populated areas, like the more publicized case of Breonna Taylor killed under parallel circumstances in 2020 (26).

Atatiana Jefferson was a 28-year-old woman from Ft. Worth, Texas playing video games with her 8-year-old nephew, Zion, late one evening in October 2019. Her neighbor saw her front door open and called the non-emergency police line to do a welfare check (27). When two officers walked to the back of the house, Atatiana grabbed her gun in fear, thinking someone was trying to break in (28). Officers saw her through the window with a gun and shot and killed Atatiana in front of Zion. They never announced their arrival nor properly identified themselves (29). Atatiana is the direct victim of officers who, in certain neighborhoods—perhaps in fear or simply “trigger happy”—do not see people but see moving targets without fear of consequences for harming.

### Witnesses

Current conceptualizations and studies will account for the acute and chronic health impacts from indirectly or vicariously experiencing police violence via media exposure or being in community with a direct victim. Yet, there are often other people at the scene of police violence, both as witnesses and (indirectly) involved parties who face direct impacts. These individuals may also sustain physical and psychological injuries, but their experiences are often absent from the discussion while the focus is on the primary victim of the police encounter. Witness physical injuries can be minor, take months of recovery, or be permanent, creating a group of people with newly developed disabilities and complex trauma. Accordingly, the associated healthcare costs can range from one-day visits to complex surgical interventions, recurrent physical and mental health provider visits across the life-course, or the need for specialized care.

In Marquita’s case, her infant son, Nai’ere, was left with permanent brain damage. A decade after her death, Nai’ere still has some difficulty with speaking and some left side paralysis that impacts his mobility, causing him to walk with a limp and at times requiring him to use a wheelchair (30). In Seyaira’s case, her grandmother, Janice, had injuries so severe she spent 3 months hospitalized before being sent to a rehabilitation facility. She is permanently disfigured and has difficulties performing basic care activities. The three children in the car were also hospitalized for months with severe injuries, while the oldest, the 15-year-old, was left with permanent physical deformities, reduced cognitive abilities, and became a wheelchair user. Her medical bills alone eclipsed $700,000 (31).

These injuries not only have medical costs, but educational and employment opportunity costs. The 15-year-old injured in Seyaira’s case was unable to return to school, meaning her educational trajectory was interrupted. For Nai’ere, his disability shapes *how* he attends school given the types of accommodations he may need. Young people with either physical or psychological trauma likely miss more days of school, meaning reduced instructional time and potentially reduced quality of education if their needs cannot be adequately accommodated. Returning to Seyaira’s case, her grandmother Janice was only in her 40s at the time of the crash. While there are not publicly facing details about what happened to her, based on her known injuries we can think about whether she had sufficient or any medical coverage, and whether her injuries resulted in job loss and thus loss of medical and other benefits. Depending on the permanence of her injuries, she may never re-enter the workforce or be limited in future employment prospects, creating compounding financial setbacks over time that likely cannot be recovered.

Beyond physical injuries, there are accompanying psychological scars that result from this violence. In addition to the witnesses with a direct relationship to the primary victim, there are also unknown numbers of bystanders, whose trauma and grief are rarely captured in public health discourse. James Smith, the neighbor of Atatiana who called the police in earnest, lives with a great deal of guilt for inciting the events that transpired. He stated, “I’m shaken. I’m mad. I’m upset. And I feel it’s partly my fault…If I had never dialed the police department, she’d still be alive” (27). Both Marquita and Seyaira were killed in public, with the potential for many drivers to see the crash in real time or pass the site of the accident. Moreover, Marquita’s death happened in a residential neighborhood, raising questions about the number of neighbors who heard or saw the aftermath of that deadly collision. Traditional frameworks systematically erase these people thereby significantly limiting our understanding of, and approaches to, the impacts of police violence on health.

Another group of witnesses usually overlooked are those implicated in causing the death. These individuals are often vilified and blamed in the mainstream media, and their involvement with the criminal-legal process leaves them unable to contest the dominant narrative. What this means is that the police version of events is often the only public-facing record of what occurred for months, sometimes years, after the encounter.

Andrew Coffee IV was the only person in the room when Alteria was shot to death. Because he fired at the police, he was arrested for attempted murder of police officers as well as for felony murder (second degree murder) of Alteria. Police and prosecutors argued that had he not fired first, they would not have returned fire and killed Alteria, making him responsible for her death (32, 33). Conversely, his lawyers argued that he fired under the assumption of an intruder and was protected under “Stand Your Ground” laws (24). A judge, however, denied this defense saying that police adequately announced themselves, despite them making the announcement while he and Alteria were asleep (34). He stood trial for felony murder of Alteria, and in 2021 a jury found him not guilty. Nevertheless, he was found guilty of illegal weapons possession due to a prior felony and was sentenced to 10 years in prison (35). Had the “Stand Your Ground” defense stood, that charge would not have been possible, and he would be free (34).

In Seyaira’s case, the off-duty officer was the sole cause for the collision. However, Seyaira’s mother, Brittany, was blamed for her death because her car seat was not adequately secured. Officers contended that had she been strapped in correctly, she would not have died (31). Brittany, who was not driving, was arrested and charged with negligent homicide. In addition, the driver and two other adults in the vehicle, including Janice discussed previously, were all issued citations for a number of driving and seatbelt violations (36). The force of the impact, and the subsequent and severe injuries incurred by the four other passengers in the car would suggest that Seyaira would have been severely injured or killed regardless: the car was flipped upside down and crushed (31). Ultimately, charges were dropped against Brittany, but the prosecutor essentially “called it even”—she was not charged for negligent homicide, but neither was the officer. Moreover, the officer was not held accountable for negligent injury to the other injured parties, nor was he even issued a citation for speeding (31).

Both Andrew and Brittany were in their early 20s when these events happened and while they were both ultimately cleared of responsibility for the deaths they did not actually cause, the public denigration of those individuals still stands without much correction or apology. They will proceed through the rest of life with these deaths publicly associated with and blamed on them. Neither of them got to voice their side of the story in as public a manner as the narrative created about them.

For Andrew specifically, he has never had the space to grieve witnessing his girlfriend’s murder, and likely has not received any mental health care for it. He has been incarcerated since the date of her murder—throughout the COVID-19 pandemic. A decade of his life will be lost to incarceration, and the lingering effects on his health and wellbeing from this trauma will remain. Given the known difficulties of trying to reintegrate post-incarceration his life’s trajectory will be forever altered. For Brittany, she had to cope with the grief of losing her baby, the severe injuries of her mother, the permanent injuries of other children, as well as the distress of being forced to navigate the criminal-legal system and the fear of potentially being incarcerated for a car accident she did not cause. It is her mugshot that primarily accompanies articles about her daughter’s death when you search for information to this day.

In neither case were the officers responsible ever held accountable, and while Andrew and Brittany navigated this quagmire of blame, they did so knowing that the people who did cause the tragedies went unscathed. Although Alteria and Seyaira may each be considered “one data point” in some datasets, all the effects on Andrew and Brittany will remain invisible in conventional public health approaches to police violence. These are all realities we call on our field to factor in when examining the effects of police violence on health.

### Survivors

The victim’s surviving loved ones—their direct biological family, chosen family, other close friends, acquaintances, and colleagues—are typically accounted for as the indirect victims of police violence in current conceptualizations. However, there are distinctive and shared experiences across these surviving groups that warrant attention for the texture they provide to the story of how trauma manifests and persists in the aftermath, aspects often hidden underneath the umbrella of “chronic stress.” The first harm next of kin withstand is the trauma of receiving the death notification.

Marquita was driving to Nai’ere’s father’s house, Dante Burgess, and was mere blocks from the destination when the crash happened. Dante endured receiving news of the crash, rushing to the main hospital where Marquita was and being informed she had passed, and then rushing to the children’s hospital to be by his son, only to be told he would never walk or talk again (30). Yolanda and Alexus Woods first received a phone call from a family friend alerting them that a shooting had happened at Andrew’s home, but they spent the better part of the morning being misled by police that Alteria was still alive. They were directed to the jail to go pick her up, and then went to the hospital to see if she was there. Ultimately, a family friend discovered Alteria had been killed through a Sheriff’s Office Facebook post and had to break the news to Yolanda and Alexus. Next of kin are also then tasked with requesting secondary autopsies if there are concerns about the first, and dealing with funeral arrangements, estate planning, and potential legalities around custodial arrangements, all of which bear significant financial and logistical burdens.

Victims played a role across multiple contexts, and their absence leaves critical gaps for others to fulfill–many of whom may lack the resources to do so effectively. This can include shifts in caretaking responsibilities. Marquita was Nai’ere’s primary parent thereby requiring someone else, potentially the other parent (Dante), older siblings, or a grandparent, to fulfill that role in her absence, along with the additional challenges associated with his new disability. Although Seyaira was the decedent, Janice (her grandmother) and the other children were also victims of non-fatal negligent police violence. Janice’s severe injuries required professional caretaking; she entered a rehabilitation facility after being hospitalized for 3 months (31). The other surviving children had temporary and permanently increased care needs that likely required their parents to call on extended relatives and community for assistance.

In Atatiana’s case, she was essential to managing pre-existing health needs within her family. Both her mom, Yolanda Carr, and older sister, Amber, dealt with heart problems. The night she was killed, she was watching Zion (one of Amber’s sons) because both Amber and Yolanda were hospitalized (37). Less than a month after Atatiana’s murder, her father, Marquis, died from a heart attack with no history of heart problems. People close to him suspect it was due to the grief of losing his only child (38). In early 2020, 3 weeks after the officer who killed Atatiana was indicted, Yolanda died (39). Finally, in June 2023, Amber also died from congestive heart failure (40). This case exemplifies how police violence is a form of Black familial destabilization and destruction. Within 4 years, a close-knit unit of siblings—Atatiana, Amber, Ashley (the oldest sister), and Adarius (the only brother)—was shattered. Ashley and Adarius experienced the loss of their youngest siblings and mother, Zion witnessed his aunt’s murder, and both he and Zayden (Zion’s brother) will grow up without multiple maternal figures (mother, aunt, and grandmother).

Across these cases determining a new structure for caregiving and parenting is far more complex when considering the long term and broad cascade of harm over time. Moreover, those who step in to fill this role may threaten their own economic stability. Chronic absence from work and using up paid time off (if available) undermines their earnings and savings. It could also precipitate job loss and access to benefits while medical and legal bills are still being incurred. Moreover, it is always a possibility that there are no next of kin or close community able to step up to the degree needed or at all, meaning a possibility of surviving parties experiencing some level of neglect.

Likewise, the adverse childhood experiences (ACEs) for children as witnesses and survivors are compounding yet remain unattended to in traditional conceptualizations. Children and adolescents are forced to grow up faster and may feel an immense pressure to take on adult responsibility. Zion’s mother said that he felt “responsible to fill the whole role of his aunt, and he has the weight of the world on his shoulders,” (29). Alexus Woods who was 16 when Alteria was murdered said she did not feel that she could grieve in public because she had to be strong for her mother. Moreover, she did not have much social support. Going back to school in the weeks following Alteria’s murder she recalls how her classmates did not speak to her but talked about her: “You could feel the whispers, or you could…walk in a room and it gets quiet.”

Survivors are also left to attend to the work of getting justice, which may never happen, and is a slow, time-consuming process. Alteria’s mother, Yolanda, spent years organizing protests to keep the focus on getting indictments against the officers. After blame was placed on Andrew and the police were not indicted, her only recourse was to file a wrongful death lawsuit (41). Meanwhile, 3 years after Atatiana’s murder, a now 11-year-old Zion was forced to take the stand in the murder trial against the police officer (42). Their family also filed a wrongful death suit. Finally, Dante Burgess simply wanted an apology for the officers’ recklessness and endangerment that killed Marquita and injured Nai’ere; it never came (30).

Most cases of police violence barely make the news while others are sensationalized. For all the surviving relatives of the cases we highlight, they were confronted with the challenges of negative and biased media portrayals. Across the board, the framing and discussion points were largely driven by the police with little fact checking with the family or other involved parties. The way journalists discuss these cases can fan the flames of hurt and trauma. For the Woods family, the media unquestioningly circulated a mugshot of Alteria that police released during their press conference discussing the raid that killed her. Despite Alteria being the victim, and the mugshot being related to a juvenile incident she was never charged with, the community was primed to blame Alteria for her own death. Journalists are quick to act but slow to issue corrections as prominently as their disinformation. Despite journalists personally apologizing to the Woods family, retractions were never made, leaving the public unaware of how they were being manipulated.

### Localized community

While the spatial impacts of police brutality have been noted in terms of spillover effects on the community and direct effects from protests, there are components to community and collective impacts that deserve more attention. Some of these are quantifiable, such as the exorbitant costs of infrastructural damage and litigation. Beyond the safety issue that vehicular chases pose to other drivers on the road, there is an uncalculated total cost of the damage that other vehicles sustain either from getting into accidents trying to avoid the chase or being hit by either the fleeing party or the police. Even when there are no bystanders, when a car under pursuit gets into a single-car accident there is damage to whatever structure that person ultimately hits—telephone poles, buildings, barricades—which all must be repaired.

For home raids, such as the one that killed Alteria Woods, broken doors and door frames can cost around $5,000 to repair, while the cost of hazardous waste cleanup after using flash bang grenades can exceed a quarter of a million dollars (43). Residents are faced with replacing windows, patching bullet holes, and managing damage to furniture and other household items that home insurance policies may not fully cover (43). Although this is a more individual-level cost, police do raid the wrong homes (43), and when individuals try to get restitution through the courts, this becomes a cost borne out by the community.

Litigating police violence is a multifaceted expense, from the municipally budgeted expenditures paying for city and state prosecutors as well as court fees related to police accountability, to the extraneous expenses of appointing special prosecutors or private lawyers to defend municipalities against police misconduct suits (44). The payout of wrongful death lawsuits come from city budgets rather than police coffers, such as the $3.5 million Atatiana’s family won in court (42). While these are just a few examples, these costs accumulate substantially and impact the entire community. Over a five-to-10-year period, the cost to litigate police misconduct has cost major cities such as Dallas, Denver, Los Angeles, and New York City anywhere from $6 to $350 million dollars (45), with the Burge police torture trials alone costing the city of Chicago $210 million, half of which went to settlements and reparation (44). This is money that is otherwise not being reinvested into life affirming systems and structures.

While the monetary costs are noteworthy, the more intangible costs likely hold greater significance for health long-term. Repeated violence from police actions and neglect violates community trust and decreases community cohesion, ultimately reducing safety and faith in institutions. The high-speed chase that killed Marquita was in a residential neighborhood with the potential to hit other pedestrians, young and old, simply doing mundane activities like walking the dog or playing in the front yard. This type of aggressive policing could lead community members to rethink whether it is safe to engage in routine activities that promote health and relationship building between neighborhood children and adults.

As mentioned earlier, none of the officers responsible for killing Seyaira or Alteria were held accountable, and blame was instead placed onto civilians. To accomplish that feat, the media collaborates in part with police public relations to give validity to alternative narratives. The victim-blaming and police exonerating discourse from police-public relations and the media can also destroy community ties, with individuals turning on each other rather than pointing the rightful finger at the institution who incited the violence. Yolanda Woods recalls the alienation she faced and cruel words she received from members of her community who dogpiled blame onto Alteria and even blamed her parenting skills:

*“In the beginning, I was angry all the time because of how nasty people were, how heartless they were. From all the nasty comments: You know, ‘she lives in Gifford,’ which is a predominant Black community ‘who cares.’ ‘Nobody cares about her because she comes from Gifford.’ ‘You lay down with dogs you get fleas.’ ‘Why was she [Alteria] there?’… ‘She [Yolanda] shouldn’t blame the police; she should blame herself for allowing her daughter to hang around people like that family.’”*

The stigmatized nature of dying through police violence given its association with presumed criminality can sow doubt in the minds of neighbors and friends as to whether a wrongful death is a cause worth rallying behind, leading to further isolation and despair.

Yolanda organized rallies on behalf of Alteria but found community support lacking. She was accused of making the community more unsafe because she ended up working with organizing groups from outside of their community. The reality was more sinister and a facet of the aftermath of police violence rarely highlighted. Police will actively engage in tactics designed to prevent community mobilization. In Alteria’s case, the Sheriff’s department ramped up traffic stops for minor violations as a form of harassment, specifically targeting residents who were protesting alongside the family:

*“Intimidation is the biggest thing here for the Sheriff department in the Black community. They are intimidating them to the point to where like in our case…they don't want any involvement with me at all, with my family at all, anything that we did they don't want anything to do with it…I did a petition when Alteria was first murdered, so we could have an outside investigation. People was refusing to sign it, that's how scared they were. They were refusing to sign the paper.”*

In addition to weaponizing the media for the purpose of character assassination, police abuse their power to harass communities that are already hyper-surveilled resulting in a level of division, tension, fear, and hostility rarely incorporated into current frameworks of the psycho-social impacts of police violence. Moreover, interference with collective action against police violence is a form of repression that only further entrenches this type of violence in racialized communities. It also serves to normalize police violence more broadly, making it more difficult to generate the type of collective outrage necessary for ending these practices.

### Broader community

The confluence of each aspect discussed previously can lead to a loss of trust in and engagement with institutions broadly. Police violence has already been tied to increased mistrust in medical institutions and the criminal legal system writ large (46). It becomes clear how that mechanism functions with the amount of interaction victims, witnesses, and survivors have with various systems leading to police violence and in its aftermath. Although not exemplified with these four case studies, victims may have initially been exposed to police contact due to the efforts of collaborating systems. For example, mental health providers finding a patient unruly, or an educator deeming a student uncooperative can lead to the police being contacted. In the aftermath of police violence, witnesses and survivors (sometimes the broader community), are forced to interact with the criminal-legal system, the health care delivery system, their respective workplaces, social service agencies, places of education, and the media. While these institutions can offer helpful and empathetic services, they can also exacerbate harm through lack of recognition of the compounding and cascading effects police violence has on health and wellbeing, which our framework aims to elucidate. Until there is greater recognition of the myriad health implications and needs across time and communities, the longstanding cycle will continue leaving people less likely to engage with or seek out assistance even when wanted or needed.

### Time and opportunity

We highlight time and opportunity costs separately from what was discussed in prior sections, as many are immeasurable and most are overlooked. For the direct victim, there is the time they lost to merely exist—their wrongful death. They had children they will never see grow up, along with many missed “firsts”—steps, days of school, kisses, dances, degrees, jobs, and partners. They had dreams unrealized—children and relationships hoped for as well as professional aspirations. Marquita was planning to return to work after spending the first 2 years of Nai’ere’s life with him at home (30), Alteria planned to attend pharmacy school (41), and Atatiana planned to go to medical school (29). Seyaira, unfortunately did not live along enough to develop dreams of her own.

The converse is true for survivors. There are children who will hit many milestones and roadblocks without a parent or caregiver there to congratulate or console them. Many friends and siblings will miss out on years of shared laughter, tears, joy, and grief. There is the time spent in bereavement, from the days spent unable to get up and function to the days spent persistently but slightly distracted. There is also the time spent thinking about what should have happened, how it could have been different, and hoping for a just outcome.

Advocacy for justice is also time consuming, from organizing and attending protests, to attending court, filing lawsuits, circulating petitions, and meeting with various officials and leaders. There is also the time it takes to rectify tarnished images and memorialize decedents. It took 4 years for Zion’s family to be awarded their wrongful death lawsuit (42). Neither Alteria’s nor Marquita’s families were successful in their civil suits against police; both were dismissed under qualified immunity (47, 48). Eight years after Alteria’s death, the Woods family is still fighting for civil liability, and their wrongful death lawsuit is on appeal (49). In the meantime, Yolanda Woods created the Alteria M. Woods Memorial Foundation and filmed a documentary to preserve her legacy and fight back against early media disparagement, and Atatiana’s siblings started a non-profit in her name and honor her through “Tay Day” (50), an annual parade to commemorate her life. Families can spend a decade or more of their lives advocating for their own cases as well as supporting others who have faced similar circumstances.

Witnesses and survivors will also spend time recovering from physical injuries, in therapy or support groups for their mental health, and attempting to rebuild depleted savings accounts and paying off associated medical and legal debts. They also may lose time to incarceration if implicated, as well as time spent trying to overcome the associated social and financial costs to being involved in the criminal-legal system, including repairing the fractured relationships with family and friends and rebuilding trust within their communities. Time cuts across everything and though it could be measured in the collective number of days, months, or years lost to any of the aforementioned activities, it is also an intangible measure of emotion and energy. Alongside the departed, it is one of the only things that cannot be restored.

## Discussion

**Figure 2:**
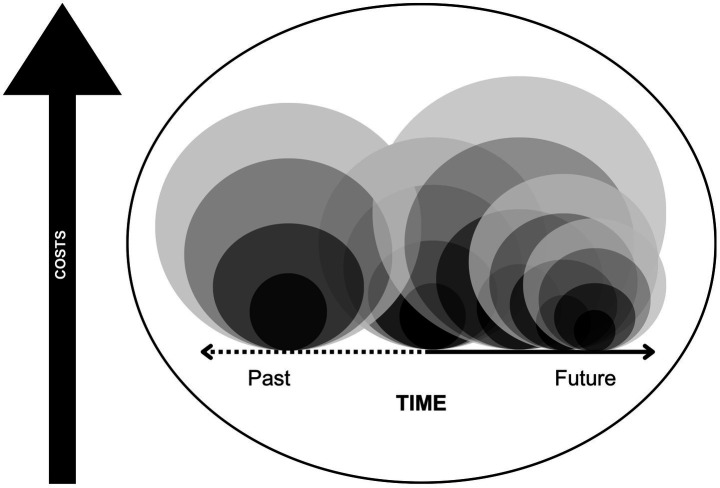
Modified conceptual framework depicting the role of frequency of police violence in sustaining harm across time.

Our conceptual framework demonstrates how one incident of police violence functions much like an earthquake. Those closest to the epicenter are most directly devastated, but the damaging shockwaves have expansive reach. While our earlier figure only included three points of police violence, Figure 2 more realistically depicts how repeated incidents layer and accumulate across time and sub-community. Each new instance of police violence becomes a different community’s epicenter, while also functioning as an aftershock for those previously at the epicenter of police violence, reactivating all their past trauma. In this way, police violence functions less like separate events and more like one continuous arc of violence that ebbs and flows in intensity. When there is little time between distinct events then there is little time for repair and restorative action, leaving people more vulnerable to the next event.

While the Woods family was still grieving and fighting for justice for Alteria, both George Floyd and Breonna Taylor were murdered. The international mass mobilization against police brutality made its way to Gifford, FL, Alteria’s hometown, resulting in days of well-attended rallies in support of Floyd and Taylor. For the Woods family, however, this reopened an old wound and generated resentment that these same people would not stand up with them just 3 years prior. Although the community finally acknowledged Alteria amidst those rallies, and invited Yolanda and Alexus to speak, they never apologized for their initial ostracism.

The continuous nature of police violence gets obscured when it is oversimplified as single, isolated data points instead of multiple people navigating a dynamic reality of implications to the incident that transcend contexts and time. While someone may survive their “earthquake,” it may be 2 years of aftershocks later, or two decades worth, that they finally succumb to the accumulative stress of this violent ecosystem. Erica Garner, the daughter of Eric Garner who infamously stated, “I cannot breathe” while dying from a chokehold by New York City Police in 2014, spent years advocating for justice on his behalf. She died prematurely at 27 after suffering a heart attack, leaving behind two young children, continuing the process of familial destruction in the wake of police violence (16). Importantly, the aftershock may not be a new act of violence, but rather a new point in time where a victim is revictimized posthumously—such as when a pivotal moment in the investigation happens or a court decision is announced reviving media attention that generates hundreds or thousands of comments and opinions.

Although this was discussed with deaths at the center of subsequent impacts, there are an exponentially greater number of non-fatal violent episodes that carry costs at differing magnitudes occurring simultaneously to the deaths we know about. Furthermore, it is a false assumption that deaths produce the greatest magnitude of harm. Though the finality of death is extreme, people who directly experience sexual, non-fatal physical, and psychological police violence are forced to live with those scars and relive memories every day in ways that public health research has not adequately accounted for. This framework can provide a starting point for thinking through and addressing additional experiences of police violence, especially as it comes to living with permanent disabilities and traumas.

### PHCRP-guided implications and recommendations

Regarding police violence, public health professionals are conditioned to list out one specific “exposure” and examine how it relates to a set of (pre-determined) health outcomes, measured in a specific and often oversimplified way. Examinations are usually limited to one individual and draw on analytic methods that are disconnected from the totality of how lives are impacted on a community level and over time. Furthermore, public health traditionally addresses health outcomes separately (e.g., policing and birth outcomes, or policing and mental health), when in fact these outcomes operate concurrently and multiplicatively, exacerbating one another. Traditional epidemiologic approaches limit us toward believing that we are confined to what will run in a statistical model with the software programs made available to us at the time. Importantly, public health’s over-reliance on quantitative methods has systematically undermined the voices and accounts of those directly impacted, which has perpetuated our flawed understanding of these dynamic processes and contributed to a normalization of this practice—ultimately bolstering a process riddled with erasure and dehumanization. Our framework addresses these gaps and applies the PHCRP to inform recommendations for moving forward in confronting the real harms of police violence.

First, the PHCRP unambiguously centers the ordinariness of racism (11)—if policing is a racial project, it is also a routine function and an expectation within the lives of those hyper-surveilled. It is presumptuous for researchers outside of communities under scrutiny to decide that high-profile police violence will be most impactful to those within the community simply because it is particularly egregious to us. This discounts the multitude of concurrent and past events that were not sensationalized in the news but still devastated people most proximal to the victims. It is not the extremity of one event that produces the health outcomes but the cumulative impact of policing across the life-course. George Floyd may have been the breaking point for one person and the point of consciousness formation for another.

Moreover, it is not just formal policing that makes up the water of conditions that shape reactions to what we define as police violence. It is also the multitude of ways that minoritized populations are policed—the way neighbors, coworkers, and the community at-large self-deputize to monitor the actions and movements of Black and minoritized people. It is how the deaths of Trayvon Martin and Ahmaud Arbery are typically included in the mental picture of what constitutes police violence despite their murderers being regular citizens. It is the way that citizen participation in neighborhood watches and digital hypersurveillance (e.g., security cameras) function much like police do, and shapes how people experience formal police violence, whether it registers as something sensational or routine (PHCRP Focus Area 1: primacy of racism).

The motivation behind this paper was to offer a reconceptualization of policing to guide critical, timely, and nuanced approaches toward not only examining, but more importantly addressing the health implications of policing on a community- and population-level. We emphasize the need for critical approaches, centering voices at the margins who are otherwise systematically omitted from epidemiological discourse and analysis on policing, with the goal of disrupting dominant practices. Efforts to center people’s stories run the risk of resulting in extractive research practices, and great care and intentionality must be taken to avoid exploiting people for their trauma. Thus, we relied heavily on news reports to piece together many of these stories and only interviewed relatives after establishing a relationship. Yet, even using secondary sources presents challenges, as journalists engage in the practice of amplifying statements from police via direct quotes, while subsequently minimizing the voices of victims and other impacted parties by paraphrasing their statements instead of including them as direct quotes (18). We therefore call on public health professionals to employ approaches that are multipronged, triangulating from several sources instead of reliance on one or another dataset to address these systematic biases in the data. (PHCRP Focus Area 2: Knowledge Production).

The framework we offer compels public health professionals to rise above employing standard epidemiological approaches and analyses at face-value, and to more deeply contextualize each data point as one linked to multiple lives (and deaths) across community contexts and time. Moreover, much of what is lacking in traditional quantitative analyses is a socio-political grounding in the relationships being analyzed. In other words, the overemphasis on “big data” (i.e., large sample sizes) has been at the expense of fully understanding and acknowledging the processes underlying patterns in population health–and most importantly has decentered the human element in the process. By more strongly incorporating qualitative approaches to this work, we can bridge a gap between the research process and the people being impacted. A significant advantage here is the opportunity to delve deeper into individual stories and highlight how multiple co-occurring dimensions of oppression dominate policing in the US—a concept aligned with the field’s growing emphasis on intersectionality There will always be a place for aggregate level analyses, but uncovering and specifying the nuances of the acute and chronic trauma borne out of both hyperlocalized and hypervisibilized policing provides for the opportunity to develop better data repositories—ones that incorporate these additional key factors that drive health outcomes. (PHCRP Focus Area 3: Conceptualization and Measurement).

As healthcrits committed to work that advances equity, the driving force behind this paper was to guide efforts that transcend the confines of academia and to outright defy health equity tourism. As stated above, the goal was not to produce another conceptual framework for the sake of simply stating our position. Rather, guided by community voices, our goal is to move public health research on policing in a direction that is more aligned with the priorities and voices expressed outside of research spaces. Our conceptual framework is largely driven by concerns expressed in our own community-rooted work and voices from community activists.

As a field, we have a professional responsibility to be accountable and responsive to communities, not just to priorities delineated by funding mechanisms or to activities that promote our individual careers. Since the American Public Health Association’s 2018 statement declaring policing a public health issue (51), there has been an increase in research on this topic largely emanating from the traditional mindset that “more research is needed,” which again lacks community grounding. We are left questioning what has since been done to yield tangible improvements in the social and material conditions of communities most impacted by policing and whether any of this work has directly improved racialized inequities. If we can start recognizing that we are beyond the point of tallying the toll and counting the costs of policing, then we can focus on creating sustainable solutions to eliminate them. This includes proactively demanding that health-providing systems (e.g., health science researchers, healthcare providers and clinics) stop collaborating with death-making systems (i.e., the police and carceral system). More importantly, it means determining how health systems and individual providers can develop models centering collective care for specifically addressing police violence and devising better and sustainable bridging across the multitude of needed systems (health care, death care, disability, economic, educational, housing, etc.) as prevention and repair. These are the guiding questions that should direct the next phase of public health work on policing (PHCRP Focus Area 4: Action).

## Conclusion

Police violence is a structural problem with both historical precedent and ongoing implications for health. Policing infiltrates multiple systems concurrently to enact said violence. Our approach to examining and hopefully preventing not only the health outcomes of police violence but the cause itself (policing) must account for that simultaneity and longevity. As a field, there is potential to advance our equity-oriented principles, if we center a race-conscious approach, and actively and genuinely are in conversation with, and uplifting, those most directly impacted. We also must keep community-based strategies to resist police violence at the forefront of our research efforts. It is only then that we can thoughtfully and effectively confront the longstanding, insidious, and multifaceted ways police violence undermines health across communities, the life-course, and generations to come.

## Data Availability

The raw data supporting the conclusions of this article will be made available by the authors, without undue reservation.
